# Hippocampal Sharp Wave/Ripples during Sleep for Consolidation of Associative Memory

**DOI:** 10.1371/journal.pone.0006697

**Published:** 2009-08-20

**Authors:** Wiâm Ramadan, Oxana Eschenko, Susan J. Sara

**Affiliations:** Collège de France, Centre National de la Recherche Scientifique, Laboratoire de la Physiologie de la Perception et de l'Action, Paris, France; University of Alberta, Canada

## Abstract

The beneficial effect of sleep on memory has been well-established by extensive research on humans, but the neurophysiological mechanisms remain a matter of speculation. This study addresses the hypothesis that the fast oscillations known as ripples recorded in the CA1 region of the hippocampus during slow wave sleep (SWS) may provide a physiological substrate for long term memory consolidation. We trained rats in a spatial discrimination task to retrieve palatable reward in three fixed locations. Hippocampal local field potentials and cortical EEG were recorded for 2 h after each daily training session. There was an increase in ripple density during SWS after early training sessions, in both trained rats and in rats randomly rewarded for exploring the maze. In rats learning the place -reward association, there was a striking further significant increase in ripple density correlated with subsequent improvements in behavioral performance as the rat learned the spatial discrimination aspect of the task. The results corroborate others showing an experience-dependent increase in ripple activity and associated ensemble replay after exploratory activity, but in addition, for the first time, reveal a clear further increase in ripple activity related to associative learning based on spatial discrimination.

## Introduction

An ever increasing number of studies in human subjects reinforce the notion that sleep is beneficial or even necessary for memory formation [1]. The crucial neurophysiologic mechanisms involved are not well-understood, although there is some suggestion that spindle oscillations of 12–20 Hz, grouped by the slow oscillations typical of slow wave sleep (SWS), provide a cortical substrate for consolidation [2]–[6]. Studies in rodents have revealed SWS-related slow oscillations in the hippocampus, as well, that are transiently coordinated with neocortical slow oscillations [7]. This co-ordinated slow wave oscillation may provide a substrate favoring hippocampal-neocortical dialogue for off-line memory consolidation [8]. Within the hippocampus, high frequency oscillations known as sharp wave/ripple complexes (SPW-Rs) are associated with synchronous discharge of a large neuronal population in multiple hippocampal sites [9]–[11]. In 1989, Buzsaki suggested that the SPW-R associated bursts are initiated by neurons whose recurrent connectivity had been transiently potentiated during the preceding awake experience [12]. Several studies have since revealed that, indeed, ensembles of neurons, firing together during a particular behavioral experience, tend to ‘replay’ during the following SWS episode [13]–[18]. This activity recorded during sleep or during still wakefulness, is more likely to occur during the SPW-R events [13], [16], [19], [20]. These cells fire together at high frequencies, which should promote Hebbian plasticity, i.e. Long Term Potentiation (LTP), thought by most investigators to be a substrate for long term memory [19], [21]. Thus, the off-line activation of network ensembles fits well within the conceptual framework of current thinking about memory consolidation mechanisms. Nevertheless, the accumulating data demonstrating off-line reactivation of previously activated networks have for the most part, been limited to studies in which rats were previously well-trained to run on a track for reward. The cell ensembles that showed replay were cells whose activity was linked to the rat's spatial location on the track. Neurons recorded during the behavior and again during subsequent sleep are ‘place cells’ whose firing is associated with a specific location in the environment [22]. While two recent studies have shown that the SW-R associated replay occurs more frequently after exploration of a novel rather than a familiar environment [19], [20], there is still no evidence for ensemble replay or changes in ripple activity related explicitly to associative learning. In the present study we examine SPW-R activity during SWS episodes following spatial exploration, place discrimination and place–reward association learning.

## Methods

### Animals

Male Sprague-Dawley rats (Charles River Laboratories, Le Genest-St-Isle, France), weighing 300–350 g, were used (n = 20). The rats were housed individually, handled daily and kept on a 12 h light/dark cycle with lights off at 8 P.M. Water and food was available *ad libitum*, unless otherwise indicated. All procedures were performed following the 1986 European Communities Council Directive and Ministère de l'Agriculture et de la Forêt, Commission Nationale de l'Experimentation Animale decree 87848 (France).

### Surgery and electrophysiological recordings

Animals were anesthetized with sodium pentobarbital (50 mg/kg, i.p., initial dose with 0.1 cc supplements given as necessary) and fixed in a stereotaxic frame. Atropine sulfate (0.2 mg/kg) was administered to minimize respiratory distress. The skull was exposed and burr holes were made for the electrode placement. An electrode constructed from a 50 µm Teflon insulated stainless steel wire, (impedance ∼0.5 MΩ) was implanted in the CA1 pyramidal cell layer (AP = −3.5, L = 2.0) at a depth of 2.0–2.5 mm. The CA1 placement of the electrode was confirmed by monitoring unit activity during implantation and subsequent histological analysis. A screw electrode was placed over the prefrontal cortex (AP = +4.0, L = 0.5) for subdural EEG recordings. A second screw electrode was placed in the skull over the cerebellum and served as the reference. Signals were amplified (×1 k) by a differential AC amplifier (model 1700, A–M Systems, Inc., Carlsborg, WA, USA), filtered (1–500 Hz), digitized at 2 kHz with 16 bit resolution using a CED Power1401 converter and Spike2 software (Cambridge Electronic Design, Cambridge, UK), and stored for off-line analysis.

Recording was performed in a transparent Plexiglas box (25×25×50 cm in size). The recording boxes were located in a quiet, isolated room adjacent to the room where the experiment was carried out, and animals were habituated to the recording environment for several days after surgery, before the first baseline sessions. The rat was connected to the amplifier by a cable allowing free movement within the box. Behavior was tracked by a video camera (Quickcam®, Logitech, Moulin du Choc, Switzerland) mounted on the top of the recording box. The video image was synchronized with electrophysiological recordings. Recordings lasted for 2–3 hours between 10 A.M. and 6 P.M. during the light period i.e., when rats spend most of their time sleeping. After the last recording session rats were deeply anesthetized with pentobarbital (100 mg/kg), perfused intracardially, and brains were extracted for histological analysis.

### Behavioral procedures

After a one-week recovery from surgery, 20 rats were put on a food-restricted diet (15–20 g per day; body weight not less than 80% of free-food weight). Each rat was habituated to the recording box and the connection procedure. Chocolate-flavored puffed rice cereal (Chocopops, Kellogg's, France) was used as reinforcement and the rats were habituated to this food before training. The training procedure and apparatus was the same as that used in previous experiments in our laboratory [21]. An eight-arm radial maze, painted black, was elevated 0.6 m from the floor. A food well, 3.5 cm in diameter and 3.25 cm deep, was located at the end of each arm. Three of the eight arms were baited, the same three for every trial for an individual rat. The maze was surrounded by salient objects and images to serve as distal cues to aid animals in navigating the environment. The experiment began with two days of pretraining where the reinforcement was available throughout the maze, not just at the extremities of the arms. A two hour baseline recording session was conducted for each rat, immediately *before* the first pretraining session. Data from each individual rat from the baseline session were used for normalization of subsequent data collected during the experiment. Twenty-four hours later, the training session for ten rats consisted of five trials with an intertrial interval of 3 min. The rat was placed on the central platform of the maze and allowed free choice of visits to the alleys. After the three baited alleys were visited, the rat was returned to the waiting cage located next to the maze. A ceiling time of 3 min was imposed. In order to ensure that performance was based on the integration of extramaze spatial information, the maze was rotated on its central axis, in a random fashion, between every trial during training, thereby eliminating any possibility of using intramaze information to navigate within the maze. The number of errors was noted at each trial, errors being defined as a visit to a non-baited arm or a repeated visit. After each session, the rat was transferred to the recording box in the adjacent room, for a 2 h recording session.

A group of pseudo-trained rats (n = 5) received the same pretraining as the trained group, as described above. For ‘training’, each pseudo-trained rat was yoked to a trained rat to determine the number of alley visits on each trial. All alleys were baited, so these rats received a small reward (one chocopop) at the end of each alley visited; thus they were not required to discriminate spatial locations nor to associate a specific place with reward or non reward. Repeated choices were not rewarded, but these rarely occurred after the first session in either group. With this procedure each group received approximately the same amount of reward and, most importantly, had exactly the same amount of locomotor activity in each session.

A nontrained, food restricted control group (n = 5) was exposed to the recording boxes and Chocopops and had dietary restriction as described above; they received no pretraining or training, but were recorded each day at the same time as the trained and pseudotrained rats.

### Data processing and statistical analysis

Behavioral performance was expressed in terms of errors. The mean number of errors at each trial was noted. If the rat did not complete a trial (i.e. did not have 3 correct choices or enter 8 arms), the unvisited arms were considered as errors, as well as repeated entrances in an arm. A grand mean of the five daily trials was calculated and learning was evaluated in the trained rats by a one-way ANOVA for repeated measures, with the mean error score for each day being the dependent variable. Daily group performance scores were compared to the performance on day 1 of training and significant learning was considered to have been expressed on the days that the score was significantly different from that of day 1 (Newman-Keuls post hoc comparisons).

Electrophysiological data processing was performed using Spike2 and custom software based on the built-in script language (Cambridge Electronic Design, Cambridge, UK). Power spectra of delta (1–4 Hz), theta (5–10 Hz), and spindle (12–15 Hz) frequency bands were calculated continuously, and sleep-wake episodes were scored by visual assessment for 10-s epochs according to standard criteria [22]. Awake state was marked by the presence of low-amplitude fast activity; SWS was identified by continuous high-amplitude slow activity and regular appearance of spindles; transitions from SWS into rapid eye movement (REM) sleep were identified by a decrease in high-amplitude slow activity, increase of theta activity and presence of spindles. Behavioral states were additionally verified by video recording. A rat was considered in slow wave sleep when it had a typical body curled posture, without movement, with its eyes closed and delta activity dominating the accompanying EEG.

**Figure 1:**
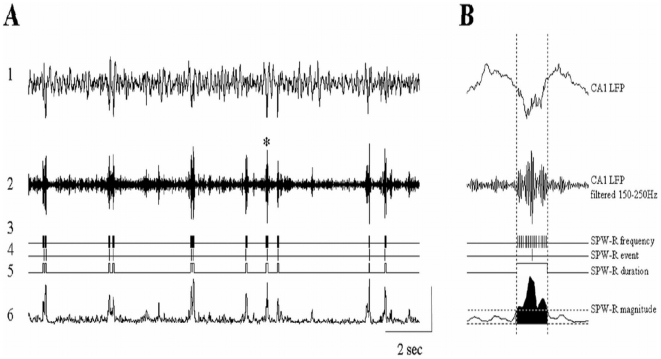
Detection and quantification of CA1 SPW-associated ripples. A: trace 1 - local field potentials recorded at the CA1 pyramidal cell layer (1–500 Hz); trace 2 - trace 1 filtered to 150–250 Hz; traces 3 – ripple troughs; trace 4 - ripple peaks; trace 5 – ripple on/off sets; trace 6 – root mean square of trace 2. B: Illustration of the ripple detection algorithm and estimation of the ripple intrinsic properties. The ripple indicated with an asterisk on panel A is enlarged. Vertical lines indicate the ripple duration (100 ms); horizontal dotted line indicates the ripple on/off set threshold; the filled region indicates the area used to estimate the ripple magnitude. The amplitude scale is 1 mV for trace 1; 0.1 mV for trace 2; 0.05 mV for trace 6.

SPW-Rs were detected by means of an automatic thresholding algorithm [23]–[24]. The hippocampal LFP signal was first filtered (150–250 Hz) and the root mean square (RMS) was calculated at every 5 ms (in a moving 10-ms window). The threshold for ripple peak detection was set to 4 standard deviations (SDs) above the mean RMS signal. The beginning and end of a ripple were marked at points at which the RMS signal dropped below 2 SD provided that these two points were separated by 25 ms; these time points were used to estimate ripple duration. For every marked ripple, the troughs were detected as the minima of the filtered LFP signal and the deepest trough was marked as the time point representing the respective SPW-R event. The magnitude of the ripple was determined by integrating the RMS signal over the duration of each ripple [21]. The ripple amplitude was estimated as a voltage difference between maximum and minimum peaks of the filtered CA1 signal during the ripple event. Ripples were detected during SWS and quiet-awake periods. (See Fig. 1 for illustration of ripple detection).

Since the temporal pattern of SWS and quiet-awake episodes and their onset and duration varied among rats, the analysis was performed for mean values over 60-min time to minimize this variability. The data were submitted to ANOVA for repeated measures, the independent factor being group (trained, pseudo-trained or control groups); the repeated factor, day of training (n = 10). *Post hoc* comparisons were made using the Newman-Keuls test, for both between group differences on specific days and session -to-session differences within groups.

## Results

### Behavior

**Figure 2:**
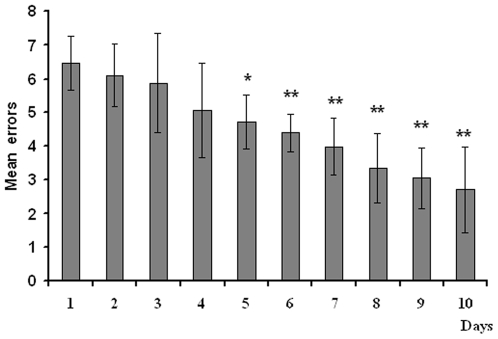
Performance accuracy in the spatial discrimination task, in terms of mean errors from 5 trials of a single session. The rat was allowed a maximum of 3 min to recover rewards from fixed locations. When all 3 rewarded arms were visited, the trial was terminated. Intertrial interval: 3 min. There was a significant decrease in errors over the daily sessions. *p<.05; **p<.01 (significant difference between each day and day 1); Values are means±SEM.

The trained group learned the task over the ten days, which included 5 massed trials per day. Learning within a five trial session was assessed in terms of mean number of errors by trial by session. One way ANOVA indicated a significant decrease in errors across days as illustrated in Figure 2 (F_9,99_ = 17.35, p<0.001). Subsequent comparisons of each day's performance with that of day 1 indicated a significant improvement in performance beginning on day 5 (different from day 1; p<.05), sustained until day 10 (p<.01 on day 10), as illustrated in Figure 2. Pseudo-trained rats were each yoked to a trained rat, such that they received the same amount of exposure to the maze on each trial, so they learned to ambulate to recover the food pellets, but did not learn the place-reward association. They also learned to avoid repeated choices, i.e. that win-stay strategies are ineffective. This was usually learned by both groups in the first session (data not shown).

### Sleep

**Figure 3:**
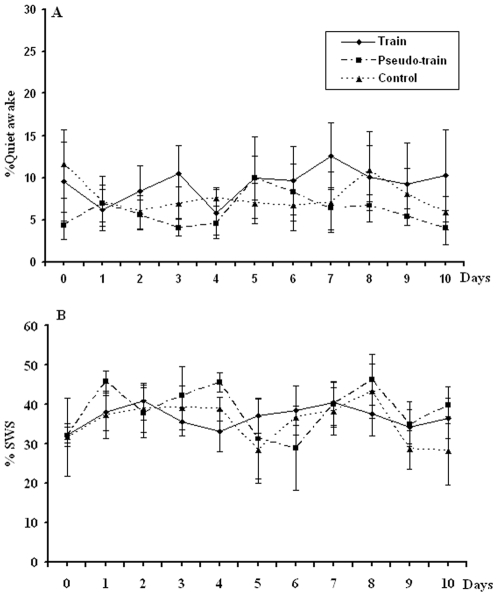
Duration of wakefulness or SWS episodes among the three treatment groups. A. Percent time spent in wakefulness (A) or SWS (B) during the 2 h recording after daily sessions in the three groups (see Methods for the treatment of each of the groups). There were no differences on either of these measures.

Training in the radial arm maze did not alter the awake-sleep pattern or total amount of post-experience SWS during the 2 h monitoring period (Figure 3). Ripples were detected during both post-training quiet awake and SWS episodes (see Methods and Figure 1). Since rats spent most of the post training period sleeping or moving, the data from the quiet awake state was sparse. Therefore, we focused on ripple characteristics during SWS. Ripple density was calculated as the number of SPW-R events/min and normalized as a percentage of the baseline ripple density recorded during sleep, for each rat, the day before starting the learning experiment. The ripple density recorded on the baseline day did not differ among the three groups: Control 36.19±3.2; Pseudotrained 37.33±2.93; Trained 33.53±51 ripples/minute. (F<1; ns).

**Figure 4:**
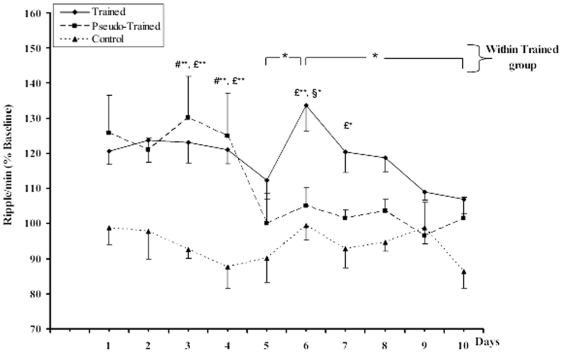
Ripple density (Ripples/min; normalized to % baseline) during the first hour of sleep after each daily session of training. Note the stable baseline in control animals receiving no training manipulation. Both “Pseudo-trained yoked rats” and “trained rats” showed a marked increase in ripple density after to the first session of training, maintained for three subsequent post training sessions. Post session ripple density in the pseudo-trained group returns to baseline by the 5th session, while in the trained group there is a significant increase in ripple density after training session 5, maintained for two subsequent sessions, returning to baseline by session 8. £: Trained vs. Control; #: Pseudo-Trained vs. Control; §trained vs. Pseudo-Trained. *p<0.05; **p<0.01. Values are means±1 SEM.

Food restricted control rats that were transported directly from their home cage to the recording session, showed remarkably stable ripple density across experimental days as seen in Figure 4. Both trained and pseudo-trained animals showed an increase in ripple density during the first hour of SWS after the first training session, compared to the control group. This increase was sustained for four training days, after which the ripple density in the pseudo-trained group returned to control level, as shown in Figure 4. ANOVA for repeated measures revealed a significant group effect (F_2,19_ = 12.7; p = .0004), a significant day effect (repeated measure) (F_9,180_ = 4.0; p = .0001) and a significant day×group interaction (F_18,153_ = 1.9; p = .017). Subsequent Newman-Keuls comparisons showed that both the trained and pseudo-trained groups show significantly more ripples on D1, D2, D3 and D4 compared to the control group (p<.01); there were no group differences on D5; the decrease in ripple density in pseudo-trained rats on D5 was significant (D5 compared to D4; p<.01). On D6 the trained group had significantly higher ripple density than *both* the pseudo-trained and the control group (p's<.01). Within group comparisons also confirmed that the further increase in ripple density on D6 in trained group, illustrated in Figure 4, was statistically significant (within group comparison D5 compared to D6; p<.01). Ripple density remained elevated in this group until D8, when there was no longer any difference from the control or pseudo-trained groups.

### Behavior-ripple correlations

**Figure 5:**
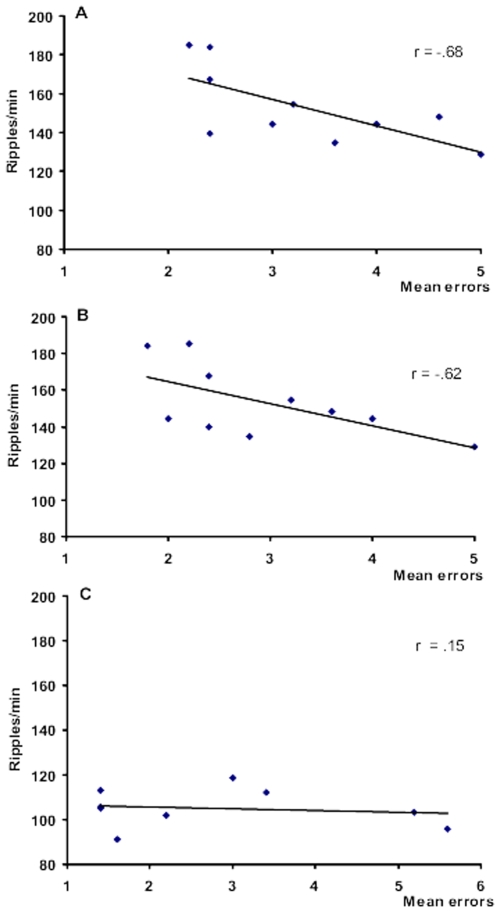
Maximum post learning ripple density, plotted as a function of error score. A. on the session following the peak ripple density. (Days 6 and 7 (n = 6) or day 7 and 8 (n = 4). B. on the following day. There was a significant correlation between ripple density (ripples/min) and the behavioural performance the following day in both A and B (p = .028 and p = .05, respectively). C. Ripple density on day 9 of training as a function of error score on day 10, when most rats had learned the task. Values are means±1 SEM.

The relationship between ripple density and behavioral measures of performance on the subsequent day was explored. The learning curve shown in Fig. 2 reflects the central tendency of the group performance over the ten day learning period, but as with all learning tasks, there are individual differences in acquisition rates, reflected by the standard errors on the learning curve. Thus to further assess the relationship between individual performance on the task and ripple density, we plotted the mean errors as a function of ripple density on the day following the highest ripple density for each rat (day 6 and 7). The rationale was that if memory consolidation was taking place during the increased ripple activity, then the reinforced memory should be expressed in improved behavioral performance on the next day. This is plotted in Fig. 5A. Since the global data indicated that ripples stayed elevated for at least two successive days of learning (day 6 and 7), a similar plot for the day following the highest ripple density and subsequent behavioral performance was generated (Fig. 5B). A linear regression analysis showed statistically significant correlations for both (r = −.68; p = .028; and r = −.62; p = .05, respectively). There were no statistically significant correlations between behavior and ripples on subsequent days when behavioral performance tended toward asymptote e.g. fig. 5C, which plots errors as a function of the previous day's ripple density, on the last training day.

**Figure 6:**
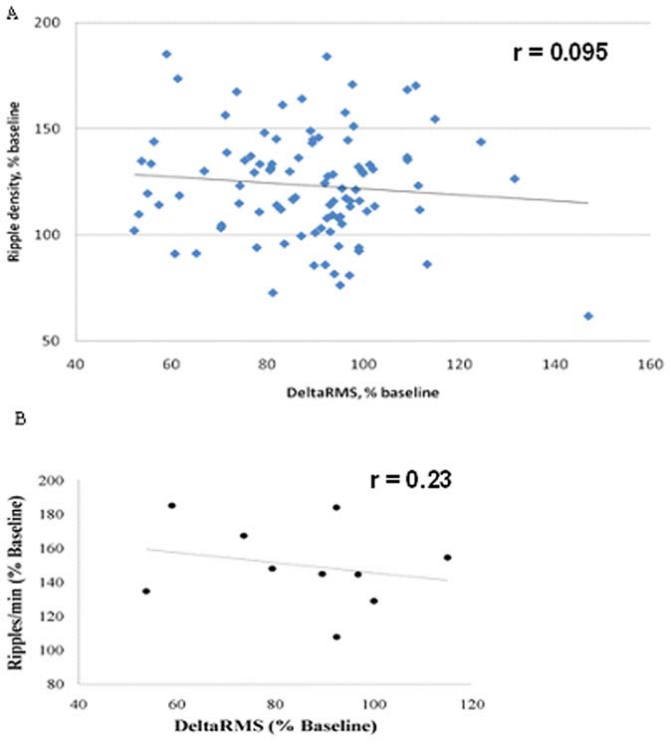
Delta power as a function of ripple density. A: Delta RMS as a function of ripple density in the trained group with data points plotted for the ten daily sessions. B: Delta RMS as a function of ripple density in the trained group during the post training session showing the highest ripple density (see Fig. 5 for determination of the sessions). The data are normalized as a percent of the baseline values recorded before training (see Methods). Note that ripple density does not vary as a function of the power of delta either overall (Fig. 5A) or on trials when there is a significant increase in ripple density. (Fig. 5B).

Delta power, considered to be an indication of depth of sleep, did not differ significantly among groups or within groups over the daily training sessions. Nevertheless, to investigate the possibility that the increase in ripple density was associated with deeper sleep, we performed a correlation analysis between ripple density and delta RMS, normalized to the baseline. The analysis was performed for the trained group, first over the whole ten day data set (Fig. 6A) and then for the training day on which the maximum increase in ripple density was observed for each individual rat (Fig. 6B). There was no correlation between the two variables for either analysis.

**Figure 7:**
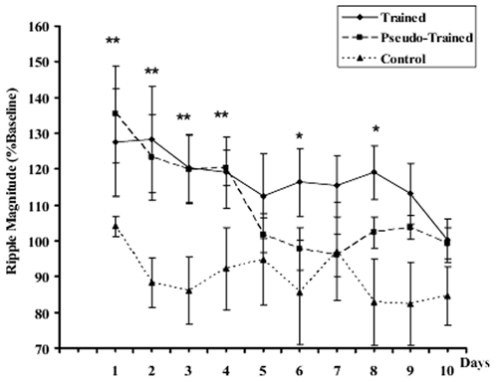
Ripple magnitude (power) normalized to baseline. There is a tendency for an overall decrease in ripple magnitude over time in the control group; while both the trained and pseudo-trained group show an initial increase, then a gradual decline, the trained group remains significantly higher through session 8. *(p<0.5) and **(p<0.01): significantly different from control. Values are means±1 SEM.

Ripple magnitude, or the power of the ripple events, was elevated in both pseudo-trained and trained groups after each of the early sessions of training. This increase was sustained over later sessions in the trained group, but returned to baseline in the pseudo-trained group, as seen in Figure 7. ANOVA for repeated measures yielded a significant effect of training day (repetition; F_9, 109_ = 2.84, p = .004); although the main effect of group was not significant nor was the interaction. Nevertheless, post-hoc comparisons suggested that there was an elevation in ripple magnitude in both groups exposed to the maze, after each of the first four sessions (trained vs. controls on days 1–4 and pseudo-trained vs. control on days 1–4, all p's<.01). In the trained group there was a tendency for this elevated ripple magnitude to persist on subsequent trials, as indicated on Figure 7 (trained vs. controls days 6 and 8; p's<.05). There was no further increase in ripple magnitude in parallel with the spatial discrimination learning, as was seen with ripple density.

Ripple duration and ripple amplitude baselines did not differ among groups (control; 96±6.8; pseudo-trained: 96±4.3; trained: 100±2.9 ss and control rat: 96±12; pseudo-trained rat: 110±11; trained rat: 109±10 mV, respectively). These measures were unchanged with respect to baseline in all three groups throughout the experiment.

## Discussion

A significant increase in ripple density during post-training SWS was seen from the first behavioral session in both the trained and pseudo-trained rats, compared to their respective baselines recorded the day before starting the training sessions, and to the food restricted control group. Each daily session consisted of five trials in which both groups were required to move about in the maze in order to obtain the rewards located at the extremities of the maze arms. Each pseudo-trained rat was yoked to a trained rat to assure that locomotor activity was the same in each group. Thus the behavior of the two groups was quite similar on the first days of training, with all rats exploring actively and consuming the rewards when encountered. The important difference between the two protocols lies in the fact that the trained group was rewarded in a fixed place, thus had to make a spatial discrimination to learn the place-reward association, while the pseudo-trained group was randomly rewarded. Significant spatial discrimination learning was expressed on day 5 of training, in that trained rats, as a group, made significantly fewer errors on that day than on the first day of training. Most importantly, there was a significant further increase in ripple density on D6 relative to D5 in the trained group, concomitant with expression of the spatial discrimination, while the ripple density of the pseudo-trained group descended to baseline in absence of a requirement for further learning. Nevertheless, it is important to note that that the exploratory activity on the maze, together with the availability of reinforcement is sufficient to drive ripples off-line, in the ‘pseudo-trained group, as others have reported.

The increase in ripple magnitude is seen after the first sessions in both pseudo-trained and trained groups Ripple magnitude remains elevated in both groups for the first four sessions, after which the pseudo-trained group returns to baseline and the same level as the control group. Ripple magnitude continues to be elevated in the trained group, sustained until after session 8, but the changes in ripple magnitude are not as robust as for ripple density and there is no further increase in this ripple characteristic as the animal learns the place-reward association on later trials. The magnitude or power of a SPW-R event has been shown to be correlated with the spiking activity of CA1 and CA3 pyramidal cells [21], [27]. Moreover, high magnitude ripples are associated with a high spatial coherence of neuronal firing throughout the hippocampus, while small magnitude ripple events are associated with more localized discharge [27]. Thus the increase in ripple magnitude early in learning suggests that additional neurons are recruited into the network that is synchronously discharging in high frequency bursts.

These high frequency oscillations that occur in the hippocampus during SWS or quiet wakefulness are thought to provide the physiological substrate for memory consolidation. The activity of the neuronal population in hippocampal CA3 area, which generates sharp waves, depends on multiple recurrent loops within CA3. The firing rate of both pyramidal cells and interneurons is maximum during the SPW-R event, but pyramidal cell firing rate increases twice as much as that of inhibitory interneurons, yielding a considerable excitatory gain [25]. This high frequency excitatory activity elicited in CA3 neurons spreads to other hippocampal areas and eventually leads to synchronous activity of a large neuronal population in CA1 [21], [26]–[28]. The population burst in CA1 will depolarize target cells and presumably promote synaptic plasticity in target cortical regions [13], [29].

The ensembles of place-tuned neurons active during behavior fire at a higher frequency during the SPW-R events in the same temporal sequence that was recorded during the behavior or in a backwards sequence [30], [31]. The replay studies have focused on ensemble activity of place cells in the hippocampus recorded in rats performing familiar tasks, requiring a high degree of stereotypic locomotor activity, such as foraging for food or running on a track. The majority of these investigators, nevertheless, suggest that the ensemble replay represents a reactivation of a memory trace established during the previous behavioral episode, even though such behavioral activities do not necessarily involve acquisition of a new information and formation of new memories. In fact, these studies do not make any attempt to evaluate learning or retention performance in relation to the replay. In a recent study we recorded ripple activity in the hippocampus after rats learned an odor-reward association task, in an attempt to address the question of the relation of the learning performance and subsequent ripples. Rats had to dig in odor-impregnated gravel to obtain food reward. One of four odors was associated with the reward. A control group foraged to find the randomly distributed reward. The group trained to dig in the presence of a specific odor to obtain reward showed sustained increase in ripple activity after the learning session, lasting at least one hour. Rats that merely had to perform the well-trained digging to obtain the reward, also showed small but significant increase in ripple density, limited to the first 30 min of SWS after the learning session [32]. Thus, rats that perform a well-trained motor response, be it running on a track, or digging in gravel, have off-line modifications in hippocampal activity, limited to the first 30 min of SWS after the behavioral activity.

In the present study, random reward in the radial maze elicits a locomotor behavior similar to that of the studies in which the rat followed a learned trajectory on a track. So the increase in ripple density and magnitude after the early learning sessions in both pseudo-trained and trained rats may be due to replay of the place cells activated during the behavioral session, as reported in previous studies. When the rat is thoroughly familiar with the routine and there is no new information to consolidate, the ripple activity returns to base line in the pseudo-trained group, i.e. after the fourth session. This result is in line with two recent reports that show greater correlated firing in place cells during ripples after exploration of a novel environment, then after exploration of a familiar environment [19], [20]. So we can conclude from those studies and our own previous and present results that mere exploration of a salient environment is sufficient to drive an increase in ripples and replay of ensembles of cells during the SWS following the behavior.

Nevetheless, there is a further increase in ripple density as the trained rats learn the place-reward association. This could be due to recruitment of other networks, afferent to the hippocampus, involved in the spatial discrimination and associative learning. There should also be an increase in release of neuromodulators such as dopamine or noradrenaline, in conjunction with the learning about reward during the training sessions, since both noradrenergic neurons of the Locus Coeruleus (LC) and dopaminergic neurons of the Ventral Tegmental Area fire in the presence of reward or stimuli that predict reward [33], [34]. Moreover, we have recently reported an increase in firing of noradrenergic neurons of the LC during SWS episodes following odor-reward association learning. These LC neurons, usually quiescent during SWS, increase their discharge rate for several minutes, to a frequency usually associated with wakefulness, while EEG remains characteristic of SWS [35]. Noradrenaline, released during this high level of LC activity, should enhance neuronal excitability and responsiveness to depolarizing inputs through action at beta-adrenergic receptors [36]. Such an action would promote or even permit neurons to fire in high frequency bursts seen during SW-R activity.

It still remains an open question whether reactivation during SWS bears a direct relation to subsequent behavioral performance. A recent study provided some support by training subjects on a visio-spatial task in the presence of a particular contextual odor. The odor was then presented as a ‘reminder’ during subsequent SWS, presumably reactivating the network active during learning. The presentation of the odor, specifically during SWS facilitated memory performance. Functional Magnetic Resonance Imaging (fMRI) showed that the hippocampus was activated during SWS by the odor presented during learning [37]. These experiments show that reminders during sleep can facilitate memory and that the reminder procedure can activate the hippocampus during SWS, but they fall short of showing a relation between activation of specific memory networks during SWS and later memory performance. Further evidence comes from an experiment in which subjects learned to navigate in a virtual town, a task largely soliciting hippocampal activity. PET imaging revealed that the same regions activated during training were activated during subsequent SWS. Most importantly, there was a positive correlation between the amount of hippocampal activity during sleep and performance on the task the following day [38]. More recent studies, using fMRI, investigated the engagement of different brain regions during spatial learning and during retrieval from remote memory. Subjects were either sleep deprived or allowed to sleep on the night after training. Sleep after training promoted an increase in hippocampal-striatum functional connectivity during retrieval. Sleep deprived subjects did not show this ‘trace reorganisation’, but nevertheless had no deficit in retention performance [39]. In a related study, using the same imaging strategy, subjects were trained on a verbal paired-associate task. Those who were allowed to sleep after learning showed a significantly higher engagement of the hippocampus during the retrieval test and simultaneous activation of the medial prefrontal regions that appeared functionally connected to hippocampus during retrieval. Even more striking was the simultaneous engagement of medial prefrontal and occipital regions during retrieval from remote memory six months later, if, and only if, sleep had occurred the night after learning. If the subject had been sleep deprived for one night, this functional reorganization was not seen at either retrieval time. Nevertheless, and quite surprisingly, the sleep deprivation had no significant effect on retention performance at 2 days or at 6 months [40].

These studies are important because they show that sleep after learning promotes functional reorganization of the memory trace with an increasing involvement of neocortical regions in long-term memory storage and retrieval. Such a dynamic view of systems level memory consolidation has been suggested by many authors [41 for review] and confirmed by animal studies [42]. The caveat is that sleep deprived subjects do not show this change in patterns of brain engagement at remote retrieval and yet show no significant retention deficit. This dissociation between post-learning, sleep-dependent, off-line reorganization of the memory trace and retention subsequent memory performance underlines the fact that we are far from understanding the spatio-temporal dynamics of systems level memory consolidation and its relation to retrieval. Nevertheless, functional imaging studies in humans, showing sleep-dependent anatomical reorganization of the memory trace, together with the present study showing an increase in hippocampal high frequency oscillations during sleep after learning, lend some support for off-line memory consolidation by network reactivation. Neither approach provides direct evidence that it is the same network of neurons active during the behavioral experience that is active during sleep. On the other hand, we did observe an additional increase in the off-line SPW-R activity as the rat learned the place-reward association and most importantly, the increased ripple activity was significantly correlated with subsequent behavioral performance.
